# Surgical correction of epispadias associated with buried penis

**DOI:** 10.1016/j.ijscr.2023.108406

**Published:** 2023-06-16

**Authors:** Tarek Abdelazeem Sabra, Sarah Magdy Abdelmohsen, Ahmed Kamel Ali

**Affiliations:** a Faculty of Medicine, Assiut University, Egypt; b Pediatric Surgery, Aswan University, Egypt

**Keywords:** Buried penis, Concealed penis, Epispadias

## Abstract

**Introduction and importance:**

Isolated male epispadias is a rare entity with incidence of approximately 1 in 120,000 live births. Epispadias usually presents with a phimotic preputial orifice where glans is not visible and hence is also known as concealed epispadias.

Buried penis in children is defined as a congenital insufficient penile skin with an unretractable foreskin that keeps the penis deep inside the pre-pubic fat. This congenital malformation of the penile envelopes is usually isolated. However, in some cases the concealed penis hides an underlying penile anomaly.

We present surgical repair of a very rare case with concealed epispadias.

**Case presentation:**

A nine-month-old infant had buried his penis, and his mother was seeking his circumcision. Local examination revealed concealed penopubic epispadias. A pediatric surgeon operated on this patient using the modified partial penile disassembly technique.

The patient was doing well at follow-up visits at one, three, and six months. There were no urethral stricture or obstructive urinary symptoms. The parents were satisfied with the cosmetic outcome.

**Clinical discussion:**

The embryogenesis and development of the urethra and the prepuce are linked. Urethral development defects (as in hypospadias or epispadias) are frequently coupled with faulty prepuce on the same side.

The goal of surgical management for epispadias is to correct the dorsal chordee and reconstruct the epispadiac urethra and glans.

Based on the cosmesis of the penis reconstruction, preservation of erectile function, and achieving urine continence, the outcome is evaluated.

**Conclusion:**

Concealed epispadias is frequently ignored because patients appear with buried penis, non-retractile prepuce, and a normal urine stream. Preoperative diagnosis and parent counseling are critical for the effective treatment of this uncommon entity. The modified partial penile disassembly procedure, in which the tunica albuginea is stitched to the pubic periosteum at 3 and 9 o'clock, can be used to correct buried epispadias.

## Highlights

• Isolated male epispadias is a rare entity with incidence of approximately 1 in 120,000 live births.
• Epispadias usually presents with a phimotic preputial orifice where glans is not visible and hence is also known as concealed epispadias.
• Parental counselling on surgery results and knowing their expectations for penile length and cosmesis are critical, and they should be counselled to have realistic expectations.
• The goal of surgical management for epispadias is to correct the dorsal chordee and reconstruct the epispadiac urethra and glans.
• Concealed epispadias is frequently ignored because patients appear with buried penis, non-retractile prepuce, and a normal urine stream.

## 1 Introduction and importance

One in 120,000 live infants have isolated male epispadias (IME), an uncommon condition [1]. Epispadias is frequently accompanied with defective dorsal penile skin, however in incredibly rare circumstances, the prepuce is intact [2,3]. Epispadias is sometimes known as concealed epispadias (CE) because it often manifests as a phimotic preputial orifice where the glans is not visible [4].

IME may be glanular, coronal, shaft, or penopubic in nature. Although it is uncommon in distal types of epispadias, urinary incontinence can develop in penopubic epispadias [5].

Children with buried penis are those who have congenitally inadequate penile skin and an unretractable foreskin that keeps the penis buried deep within the prepubic fat. The fascia and penile envelopes are often not attached to one another at the base of the penis. Usually isolated, this congenital penile envelope deformity. However, occasionally the buried penis masks an underlying penile abnormality [6,7].

The work has been reported in line with the SCARE criteria [8].

## 2 Case presentation

**Fig. 1:**
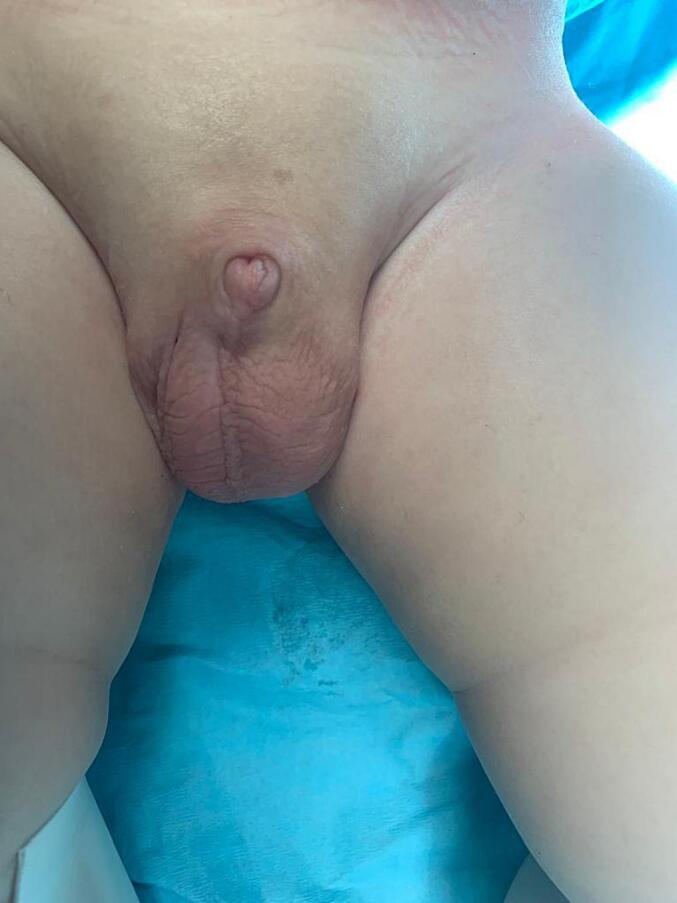
A buried penis in 9 months old infant.

**Fig. 2:**
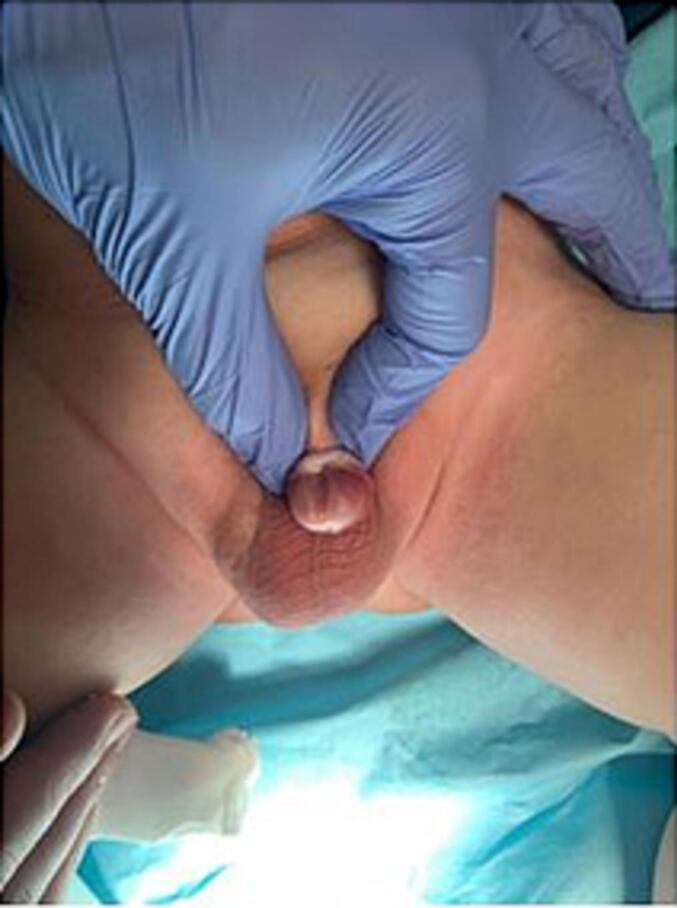
Local examination revealed concealed epispadias.

A nine-month-old infant had buried his penis, and his mother was seeking his circumcision. On physical examination, the infant was generally well, overweight but not obese. Local examination revealed concealed penopubic epispadias (Fig. 1, Fig. 2). The penis was 1 cm long and 0.5 cm wide. No chordee was detected. The scrotum was normally developed, and both testes were in the scrotum. Under general anesthesia and caudal spinal block, the boy underwent operative correction of his penile anomaly. A pediatric surgeon operated on this patient using the modified partial penile disassembly technique. An IV dose of first-generation cephalosporin was injected with anesthesia induction.

**Fig. 3:**
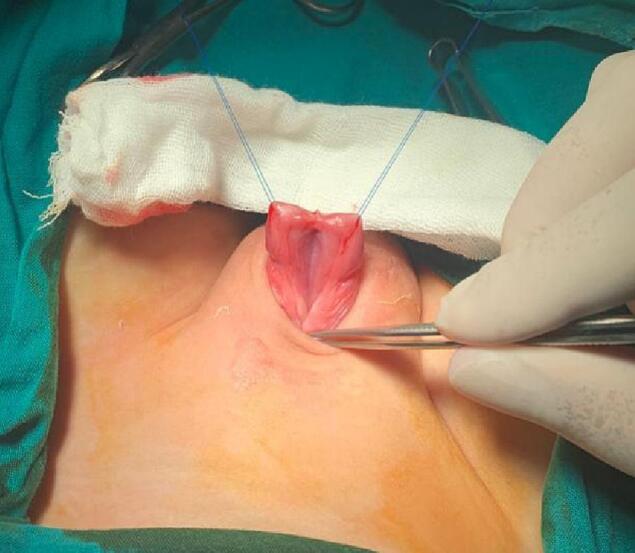
A stay suture was applied at the meatus tip.

**Fig. 4:**
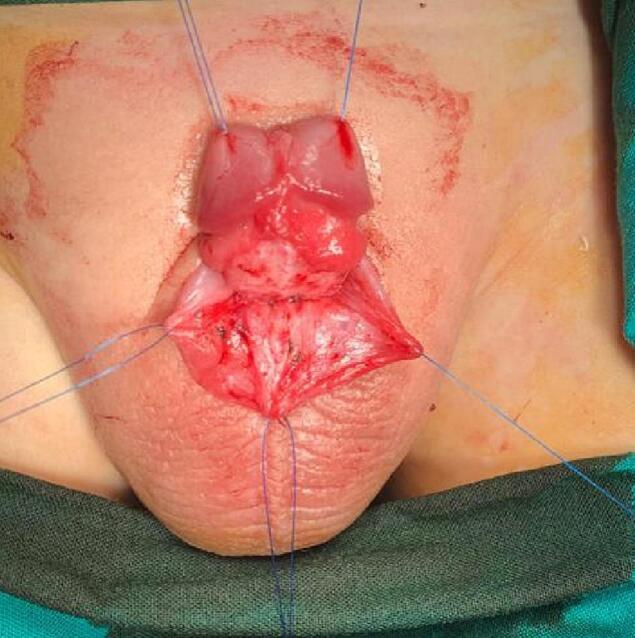
Degloving of the penile skin.

**Fig. 5:**
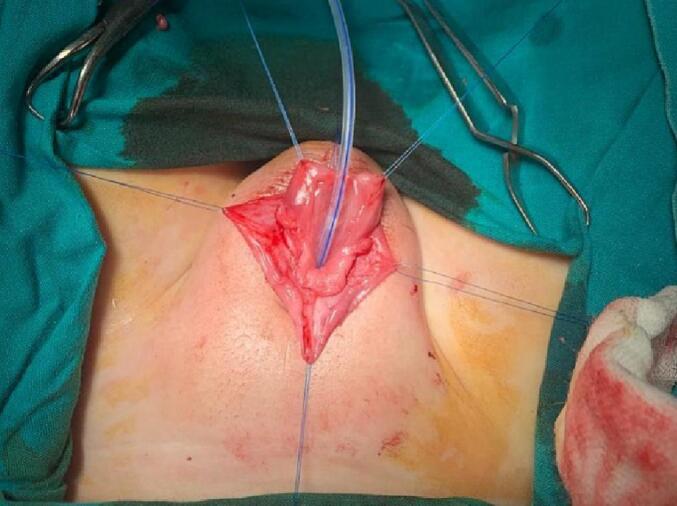
A silicon urinary catheter 6 Fr. was inserted.

**Fig. 6:**
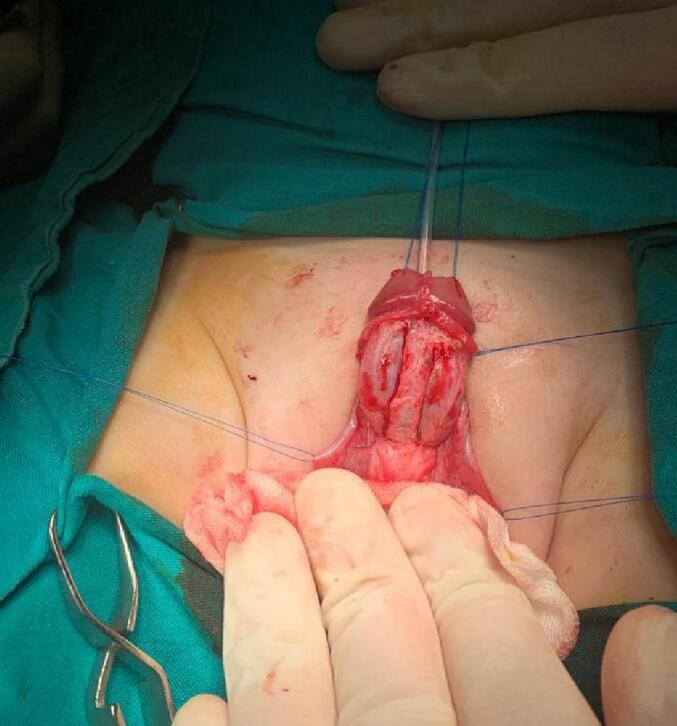
Mobilization of the urethral plate from ventral aspect.

**Fig. 7:**
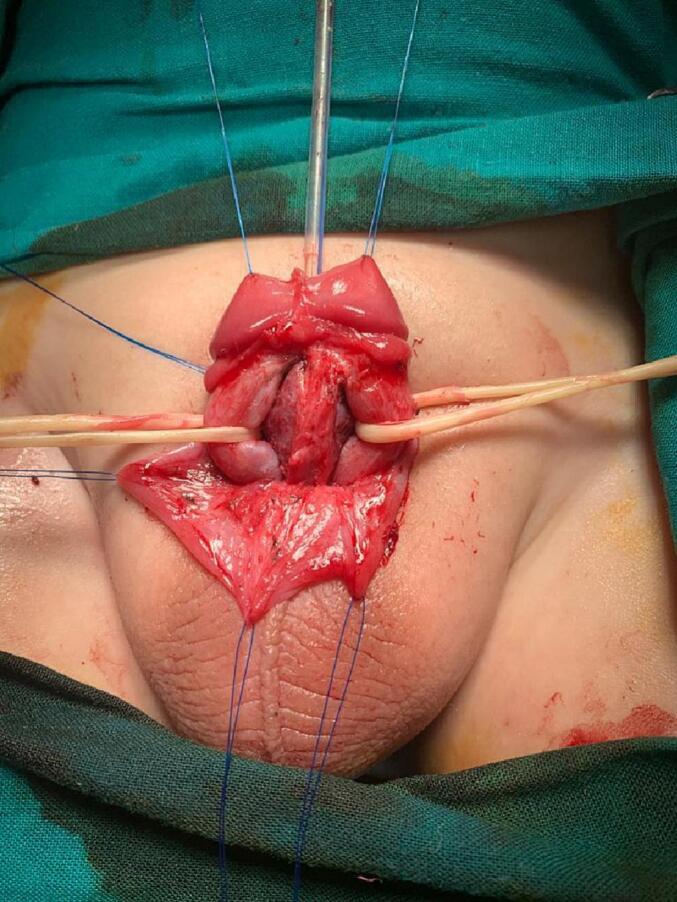
The penile shaft was separated into corpora; two corpora cavernosa (in each side with yellow rubber band) and one corpora spongiosum with its urethral plate in the middle. (For interpretation of the references to colour in this figure legend, the reader is referred to the web version of this article.)

A stay suture was applied at the meatus tip (Fig. 3). Then injection of lidocaine with adrenaline solution in a concentration of 1:100000 at the meatus tip, the margin of the urethral plate, and around the corona A circumferential incision was made around the corona and then extended down in a U shape around the urethral plate. Now the penile skin can be degloved up to the root of the penis (Fig. 4). A silicon catheter (6 Fr.) was inserted to support the urethra at the next steps (Fig. 5). We start mobilization of the urethral plate from the ventral aspect (Fig. 6). Ventrally, the dissection continues up to the membranous urethra and dorsally up to the bladder neck. At the point of the symphysis pubis, the penopubic ligament will be divided. Distally, the urethral plate was dissected up to mid-glans. The penile shaft was separated into corpora; two corpora cavernosa and one corpora spongiosum with its urethral plate (Fig. 7). If the urethra is narrow, a midline incision can be made inside the urethral plate to widen it.

**Fig. 8:**
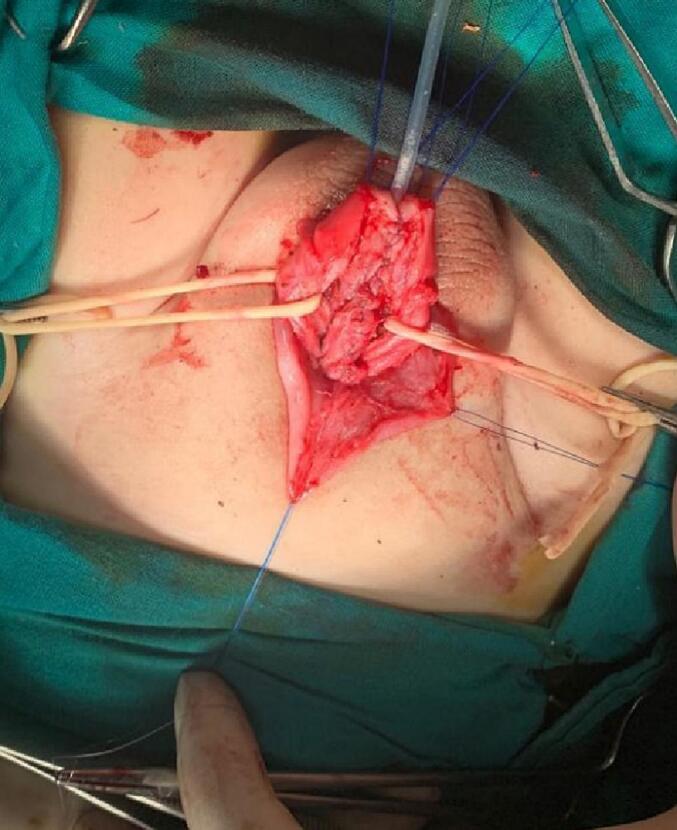
Spongioplasty.

**Fig. 9:**
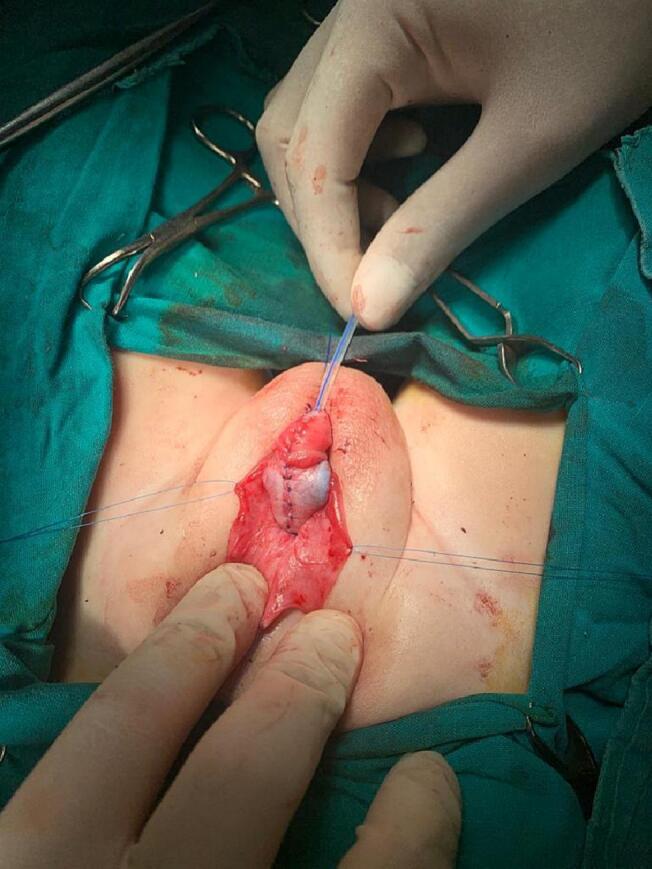
Glanuloplasty, meatoplasty and penile shaft repair.

**Fig. 10:**
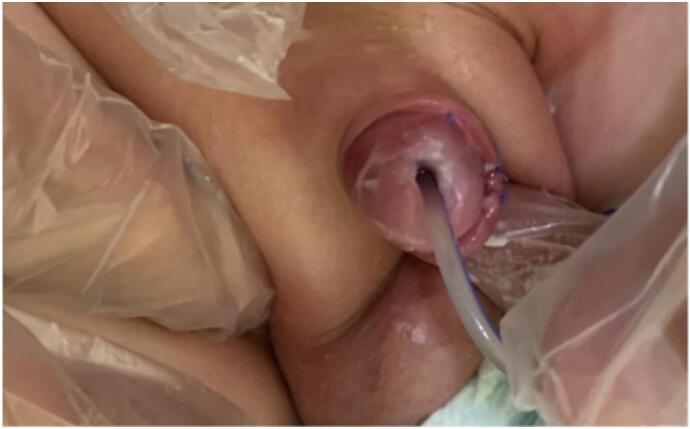
Good wound healing at two weeks postoperative.

Repairs started by spongioplasty used continuous Vicryl-5-0 sutures. Take care at this step to correct the meatus to the correct position at the tip of the glans (Fig. 8). To continue the glanuloplasty and the penile shaft, the two corpora cavernosa were repaired dorsally and the corpora spongiosum ventrally. Now the vertical-shaped meatus opening was located at the tip of the conical glans (Fig. 9). For the correction of the buried penis, the tunica albuginea was fixed at the pubic periosteum by two stitches at 3 and 9 o'clock. The ventral preputial skin was rotated dorsally to cover the skin deficiency in this area, and interrupted Vicryl 4-0 sutures were used for fixation. Closure of the wound with soft gauze filled with Garamycin cream. The operated penis was fixed to the public area in a straight position with the aid of Elastoplast. The patient was discharged from the hospital on the second postoperative day and continued the first-generation cephalosporin syrup and paracetamol for two weeks. The dressing was changed on the 5th postoperative day. The urethral catheter was removed on the 14th postoperative day. The wound looked clean with a good healing process during the follow up visits (Fig. 10). The patient was doing well at follow-up visits at one, three, and six months. There were no urethral stricture or obstructive urinary symptoms. The parents were satisfied with the cosmetic outcome.

## 3 Clinical discussion

The embryogenesis and development of the urethra and the prepuce are linked. Urethral development defects (as in hypospadias or epispadias) are frequently coupled with faulty prepuce on the same side [1].

The cloaca separates into a posterior component, the anorectal canal, and an anterior portion, the primitive urogenital sinus, during the second month of intrauterine life. The bladder, proximal prostatic urethra, and membranous urethra all originate from this urogenital sinus. The phallic cloaca, the most caudal portion of the cloaca, extends distally via the growing genital tubercle. The cloaca is displaced to the caudal part of the growing glans as a result of genital tubercle proliferation. Failure to complete this phase leads in the epispadias abnormality [1,9].

The prepuce develops during the third month of intrauterine life when the genital tubercle proliferates. After median cleavage of the urethral plate, two sets of tissue folds emerge on either side of the urethral groove on the ventral surface. The urethra is formed when the medial endodermal folds join in the ventral midline. The penile shaft skin and prepuce are formed when the more lateral ectodermal folds fuse over the developing urethra. Just proximal to the growing glans penis, an ectoderm ring arises. As the prepuce grows, this skin advances over the corona of the glans and finally covers it fully [1,9,10].

Isolated male epispadias (IME) is a relatively rare condition (1 in 120,000 males), and it is the mildest form of the bladder exstrophy-epispadias complex (BEEC) [1]. According to McCahill et al. [11] vigorous mesenchyme development between the preputial fold and glandular lamella is what causes the phenomena of epispadias with an intact prepuce, which causes the fold to move distally until it completely encloses the glans. The urethral defect and the glans are both covered if these folds develop close to the urethral defect.

Preoperative diagnosis of CE is frequently missed because patients come with buried penis, non-retractile prepuce, and normal urine stream, despite several clinical symptoms that might arouse a suspicion of an underlying condition. Dorsally directed urine stream, dorsally situated preputial aperture, dorsal chordee, gap felt between corpora cavernosa, bifid, spade-like enlarged glans, lack of penile raphe on glans, and/or absence of frenulum are all signs of epispadias [12,13]. Our patient was accidentally diagnosed with CE after retraction of penile foreskin under general anesthesia.

Parental counseling on surgery results and knowing their expectations for penile length and cosmesis are critical, and they should be counselled to have realistic expectations [13].

Although preoperative testosterone can be administered to increase penile length, its rationale is unclear [11,14]. In our case, we did not need testosterone stimulation.

The goal of surgical management for epispadias is to correct the dorsal chordee and reconstruct the epispadiac urethra and glans. The degree of epispadias can be treated surgically using a variety of methods. The urethra is positioned between the corpora cavernosa utilising the Cantwell-Ransley approach in coronal, shaft, or penopubic epispadias. The IPGAM method (reverse MAGPI) is adequate in situations of glandular epispadias to produce a successful outcome. Another choice is Mitchell's Complete Penile Disassembly Technique, which necessitates the creation of a hypospadias urethra. The surgeon chose the modified partial penile dissection approach to treat this patient due to intraoperative findings.

Based on the cosmesis of the penis reconstruction, preservation of erectile function, and achieving urine continence, the outcome is evaluated [11,15]. Compared to traditional isolated epispadias, CE has a better prognosis [11,16]. At the one, three, and six-month follow-up appointments, our patient was doing well. There was no urethral structure or obstruction of the urinary tract. The aesthetic effect satisfied the parents.

## 4 Conclusion

Concealed epispadias is frequently ignored because patients appear with buried penis, non-retractile prepuce, and a normal urine stream. Preoperative diagnosis and parent counseling are critical for the effective treatment of this uncommon entity. The modified partial penile disassembly procedure, in which the tunica albuginea is stitched to the pubic periosteum at 3 and 9 o'clock, can be used to correct buried epispadias.

## Consent

Written informed consent was obtained from the patient's parents/legal guardian for publication and any accompanying images. A copy of the written consent is available for review by the Editor-in-Chief of this journal on request.

## Provenance and peer review

Not commissioned, externally peer reviewed.

## Ethical approval

Our institution does not require ethical approval for reporting individual cases or case series.

## Funding source

The author(s) received no financial support for the research, authorship and/or publication of this article.

## CRediT authorship contribution statement

Ahmed Kamel Ali and Sara Magdy contributed to management and wrote the manuscript.

Tarek Abdelazeem Sabra contributed to the acquisition of data and revision of manuscript.

Ahmed Kamel Ali and Tarek Abdelazeem Sabra were the surgeons who operated this case. Ahmed Kamel Ali critically revised the manuscript. All authors read and approved the final manuscript.

## Guarantor

Ahmed Kamel Ali.

## Registration of research studies

NCT05852535.

## Declaration of competing interest

The author(s) declared no potential conflicts of interest with respect to the research, authorship and/or publication of this article.

## Data Availability

The data that support the findings of this study are available from the corresponding author upon reasonable request.

## References

[1] Stephens F.D., Hutson JmJJoPU (2005).

[2] Sarin Y.K., Sinha AjIJoU (2001).

[3] Maitama H., Ahmed M., Bello A., Mbibu HjAJoU (2012).

[4] Sina A., Alizadeh F.J.U.J. (2011).

[5] Levin T.L., Han B., Little B.P. (2007). Congenital anomalies of the male urethra. Pediatr. Radiol..

[6] Lardellier-Reynaud F., Varlet F., Francois M., Mouriquand P. (2011). Congenital buried penis in children. Prog. Urol..

[7] Maizels M., Zaontz M., Donovan J., Bushnick P.N., Firlit C.F. (1986). Surgical correction of the buried penis: description of a classification system and a technique to correct the disorder. J. Urol..

[8] Agha R.A., Franchi T., Sohrabi C., Mathew G., Kerwan A., Group S. (2020). The SCARE 2020 guideline: updating consensus Surgical CAse REport (SCARE) guidelines. Int. J. Surg..

[9] Sarma V.P.J.I.S.J. (2019).

[10] Perovic S.V., Vukadinovic V., Djordjevic M.L., Djakovic N.G. (1999). Penile disassembly technique for epispadias repair: variants of technique. J. Urol..

[11] McCahill P.D., Leonard M.P., Jeffs R.D. (1995). Epispadias with phimosis: an unusual variant of the concealed penis. Urology.

[12] Liu X., He D.W., Hua Y., Zhang D.Y., Wei G.H. (2013). Congenital completely buried penis in boys: anatomical basis and surgical technique. BJU Int..

[13] Garge S. (2016). Concealed epispadias: report of two cases and review of literature. Urology.

[14] Bos E.M., Kuijper C.F., Chrzan R.J., Dik P., Klijn A.J., de Jong T.P. (2014). Epispadias in boys with an intact prepuce. J. Pediatr. Urol..

[15] Bhattacharya V., Sinha J.K., Tripathi F.M. (1982). A rare case of epispadias with normal prepuce. Plast. Reconstr. Surg..

[16] Merlob P., Mor N., Reisner S.H. (1987). Epispadias with complete prepuce and phimosis in a neonate. Clin. Pediatr. (Phila).

